# Reverse Engineering Cellular Networks with Information Theoretic Methods

**DOI:** 10.3390/cells2020306

**Published:** 2013-05-09

**Authors:** Alejandro F. Villaverde, John Ross, Julio R. Banga

**Affiliations:** 1 Bioprocess Engineering Group, IIM-CSIC, Eduardo Cabello 6, Vigo 36208, Spain; E-Mail: julio@iim.csic.es; 2 Department of Chemistry, Stanford University, Stanford, CA 94305, USA; E-Mail: john.ross@stanford.edu

**Keywords:** systems biology, network modeling, data-driven modeling, information theory, statistics, systems identification

## Abstract

Building mathematical models of cellular networks lies at the core of systems biology. It involves, among other tasks, the reconstruction of the structure of interactions between molecular components, which is known as network inference or reverse engineering. Information theory can help in the goal of extracting as much information as possible from the available data. A large number of methods founded on these concepts have been proposed in the literature, not only in biology journals, but in a wide range of areas. Their critical comparison is difficult due to the different focuses and the adoption of different terminologies. Here we attempt to review some of the existing information theoretic methodologies for network inference, and clarify their differences. While some of these methods have achieved notable success, many challenges remain, among which we can mention dealing with incomplete measurements, noisy data, counterintuitive behaviour emerging from nonlinear relations or feedback loops, and computational burden of dealing with large data sets.

## 1. Introduction

Systems biology is an interdisciplinary approach for understanding complex biological systems at the system level [1]. Integrative mathematical models, which represent the existing knowledge in a compact and unambiguous way, play a central role in this field. They facilitate the exchange and critical examination of this knowledge, allow to test if a theory is applicable, and make quantitative predictions about the system's behaviour without having to carry out new experiments. In order to be predictive, models have to be “fed” (calibrated) by data. Although the conceptual foundations of systems biology had been laid several decades ago, during most of the 20th century the experimental data to support its models and hypotheses were missing [2]. With the development of high-throughput techniques in the 1990s, massive amounts of “omics” data were generated, providing the push required for the rapid expansion of the field.

This review paper deals with the problem of constructing models of biological systems from experimental data. More specifically, we are interested in reverse engineering cellular systems that can be naturally modeled as biochemical networks. A network consists of a set of nodes and a set of links between them. In cellular networks the nodes are molecular entities such as genes, proteins, or metabolites. The links or edges are the interactions between nodes, such as the chemical reactions where the molecules are present, or a higher level abstraction such as a regulatory interaction involving several reactions. Thus cellular networks can be classified, according to the type of entities and interactions involved, as gene regulatory, metabolic, or protein signaling networks.

The main goal of the methods studied here is to infer the network structure, that is, to deduce the set of interactions between nodes. This means that the focus is put on methods that—if we choose metabolism as an example—aim at finding which metabolites appear in the same reaction, as opposed to methods that aim at the detailed characterization of the reaction (determining its rate law and estimating the values of its kinetic constants). The latter is a related but different part of the inverse problem, and will not be considered here.

Some attributes of the entities are measurable, such as the concentration of a metabolite or the expression level of a gene. When available, those data are used as the input for the inference procedure. For that purpose, attributes are considered random variables that can be analyzed with statistical tools. For example, dependencies between variables can be expressed by correlation measures. Information theory provides a rigorous theoretical framework for studying the relations between attributes.

Information theory can be viewed as a branch of applied mathematics, or more specifically as a branch of probability theory [3], that deals with the quantitative study of information. The foundational moment of this discipline took place in 1948 with the publication by C.E. Shannon of the seminal paper “A mathematical theory of communication” [4]. Indeed, that title is a good definition of information theory. Originally developed for communication engineering applications, the use of information theory was soon extended to related fields such as electrical engineering, systems and control theory, computer science, and also to more distant disciplines like biology [5]. Nowadays the use of information-theoretic concepts is common in a wide range of scientific fields.

The fundamental notion of information theory is entropy, which quantifies the uncertainty of a random variable and is used as a measure of information. Closely related to entropy is mutual information, a measure of the amount of information that one random variable provides about another. These concepts can be used to infer interactions between variables from experimental data, thus allowing reverse engineering of cellular networks.

A number of surveys, which approach the network inference problem from different points of view, including information-theoretic and other methods, have been published in the past. To the best of the authors' knowledge, the first survey focused on identification of biological systems dates back to 1978 [6]. More recently, one of the first reviews to be published in the “high-throughput data era” was [7]. Methods that determine biochemical reaction mechanisms from time series concentration data were reviewed in [8], including parameter estimation. In the same area, a more recent perspective (with a narrower scope) can be found in [9]. Techniques developed specifically for gene regulatory network inference were covered in [10]—which included an extensive overview of the different modeling formalisms—and in [11], as well as in other reviews that include also methods applicable to other types of networks [12,13]. Methods for the reconstruction of plant gene co-expression networks from transcriptomic data were reviewed in [14]. The survey [15] covers not only network inference but also other topics, although it does not discuss information theoretic methods. Recently, [16] studied the advantages and limitations of network inference methods, classifying them according to the strategies that they use to deal with underdetermination. Other reviews do not attempt to cover all the literature, but instead focus on a subset of methods on which they carry out detailed comparisons, such as [17–20].

The problem of network inference has been investigated in many different communities. The aforementioned reviews deal mostly with biological applications, and were published in journals of bioinformatics, systems biology, microbiology, molecular biology, physical chemistry, and control engineering communities. Many more papers on the subject are regularly published in journals from other areas. Systems identification, a part of systems and control theory, is a discipline in its own right, with a rich literature [21,22]. However, in contrast to biology, it deals mostly with engineered systems, and hence its approaches are frequently difficult to adapt or not appropriate for reverse engineering complex biological systems. Other research areas such as machine learning have produced many theoretically rigorous results about network inference, but their transfer to biological applications is not frequently carried out. In this survey we intend to give a broad overview of the literature from the different—although sometimes partially overlapping—communities that deal with the network inference problem with an information theoretic approach. Thus we review papers from the fields of statistics, machine learning, systems identification, chemistry, physics, and biology. We focus on those contributions that have been or are more likely to be applied to cellular networks.

## 2. Background

### 2.1. Correlations, Probabilities and Entropies

Biological sciences have a long history of using statistical tools to measure the strength of dependence among variables. An early example is the correlation coefficient *r* [23,24], which quantifies the linear dependence between two random variables *X* and *Y*. It is commonly referred to as the Pearson correlation coefficient, and it is defined as the covariance of the two variables divided by the product of their standard deviations. For *n* samples it is:

(1) $$r(X,Y)=\frac{\sum_{i=1}^{n}\left(X_{i}-\bar{X})(Y_{i}-\bar{Y}\right)}{\sqrt{\sum_{i=1}^{n}{\left(X_{i}-\bar{X}\right)}^{2}}\sqrt{\sum_{i=1}^{n}{\left(Y_{i}-\bar{Y}\right)}^{2}}}$$

where *X_i_*, *Y_i_* are the *n* data points, and *X̄*, *Ȳ* are their averages. If both variables are linearly independent, *r*(*X*, *Y*) = 0 and knowledge of one of them does not provide any information about the other. In the opposite situation, where one variable is completely determined by the other, all the data points lie on a line and *r*(*X*,*Y*) = ±1.

It should be noted that in this context the word “linear” may be used in two different ways. When applied to a deterministic system, it means that the differential equations that define the evolution of the system's variables in time are linear. On the other hand, when applied to the relationship between two variables, it means that the two-dimensional plot of their values (not of the variables as a function of time, *X*(*t*), *Y*(*t*), but of one variable as a function of the other, *X*(*Y*)) forms a straight line, independently of the character of the underlying system.

A related concept, partial correlation, measures the dependence between two random variables *X* and *Y* after removing the effect of a third variable *Z*. It can be expressed in terms of the correlation coefficients as follows:

(2) $$r(X,Y|Z)=\frac{r(X,Y)-r(X,Z)r(Y,Z)}{\sqrt{\left(1-r^{2}(X,Z))(1-r^{2}(Y,Z)\right)}}$$

The Pearson coefficient is easy to calculate and symmetric, and its range of values has a clear interpretation. However, as noted in [25,26], it uses the second moment of the pair distribution function (1), discarding all higher moments. For certain strong nonlinearities and correlations extending over several variables, higher than the second moment of the pair probability distribution function may contribute and an alternative measure of dependence may be more appropriate. Hence the Pearson coefficient is not an accurate way of measuring nonlinear correlations, which are ubiquitous in biology. A more general measure is mutual information, a fundamental concept of information theory defined by Shannon [4]. To define it we must first introduce the concept of entropy, which is the uncertainty of a single random variable: let *X* be a discrete random vector with alphabet *χ* and probability mass function *p*(*x*). The entropy is:

(3) $$H(X)=-\sum_{x\in \chi }p(x)\log p(x)$$

where log is usually the logarithm to the base 2, although the natural logarithm may also be used. Entropy can be interpreted as the expected value of $\log\frac{1}{p(x)}$, that is

(4) $$H(X)=E_{p}\log\frac{1}{p(X)}$$

The joint entropy of a pair of discrete random variables (X,Y) is

(5) $$H(X,Y)=-\sum_{x}\sum_{y}p(x,y)\log p(x,y)=-E_{p}\log p(X,Y)$$

Conditional entropy *H*(*Y*∣*X*) is the entropy of a random variable conditional on the knowledge of another random variable. It is the expected value of the entropies of the conditional distributions, averaged over the conditioning random variable. For example, for two random variables *X* and *Y* we have

(6) $$\begin{array}{ll} H(Y|X) & =\sum_{x}p(x)H(Y|X=x)=-\sum_{x}p(x)\sum_{y}p(y|x)\log p(y|x)\\ & =-\sum_{x}\sum_{y}p(x,y)\log p(y|x)=-E_{p(x,y)}\log p(Y|X) \end{array}$$

The joint entropy and the conditional entropy are related so that the entropy of a pair of random variables is the entropy of one plus the conditional entropy of the other:

(7) H(X,Y)=H(X)+H(Y∣X)

The relative entropy is a measure of the distance between two distributions with probability functions *p*(*x*) and *q*(*x*). It is defined as:

(8) $$D(p∥q)=\sum_{x}p(x)\log\frac{p(x)}{q(x)}=E_{p}\log\frac{p(x)}{q(x)}$$

The relative entropy is always non-negative, and it is zero if and only if *p* = *q*. However, it is not a true distance because it is not symmetric and it does not satisfy the triangle inequality.

### 2.2. Mutual Information

Mutual information, *I*, is a special case of relative entropy: it is the relative entropy between the joint distribution, *p*(*x*, *y*), and the product distribution, *p*(*x*)*p*(*y*), that is:

(9) $$I(X,Y)=\sum_{x}\sum_{y}p(x,y)\log\frac{p(x,y)}{p(x)p(y)}=D(p(x,y)∥p(x)p(y))=E_{p(x,y)}\log\frac{p(X,Y)}{p(X)p(Y)}$$

Linfoot [27] proposed the use of mutual information as a generalization of the correlation coefficient and introduced a normalization with values ranging from 0 to 1:

(10) $$I_{L}(X,Y)=\sqrt{1-e^{-2I(X,Y)}}$$

The mutual information is a measure of the amount of information that one random variable contains about another. It can also be defined as the reduction in the uncertainty of one variable due to the knowledge of another. Mutual information is related to entropy as follows:

(11) I(X,Y)=H(X)-H(X∣Y)=H(X)+H(Y)-H(X,Y)

Finally, the conditional mutual information measures the amount of information shared by two variables when a third variable is known:

(12) I(X,Y∣Z)=H(X∣Z)-H(X∣Y,Z)

If Y and Z carry the same information about X, the conditional mutual information *I*(*X*, *Y*∣*Z*) is zero.

**Figure 1.**
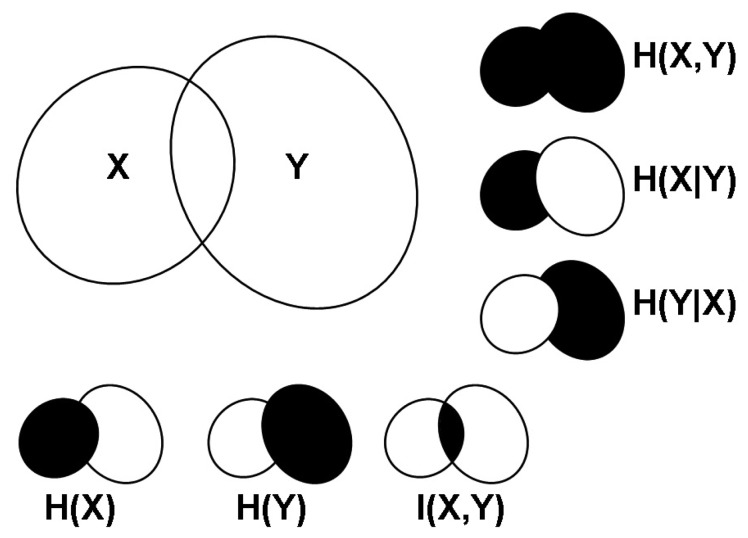
Graphical representation of the entropies (*H*(*X*), *H*(*Y*)), joint entropy (*H*(*X*, *Y*)), conditional entropies (*H*(*X*∣*Y*), *H*(*Y*∣*X*)), and mutual information (*I*(*X*, *Y*)) of a pair of random variables (*X*, *Y*).

The relationship between entropy, joint entropy, conditional entropy, and mutual information is graphically depicted in Figure 1. Note that until now we have considered implicitly discrete variables; in the case of continuous variables the Σ are replaced by ∫. For more detailed descriptions of these concepts, see [28].

Mutual information is a general measure of dependencies between variables. This suggests its application for evaluating similarities between datasets, which allows for inferring interaction networks of any kind: chemical, biological, social, or other. If two components of a network interact closely, their mutual information will be large; if they are not related, it will be theoretically zero. As already mentioned, mutual information is more general than the Pearson correlation coefficient, which is only rigorously applicable to linear correlations with Gaussian noise. Hence, mutual information may be able to detect additional non-linear correlations undetectable for the Pearson coefficient, as has been shown for example in [29] where it was demonstrated with metabolic data.

In practice, for the purpose of network inference, mutual information cannot be analytically calculated, because the underlying network is unknown. Therefore, it must be estimated from experimental data, a task for which several algorithms of different complexity can be used. The most straightforward approximation is to use a “naive” algorithm that partitions the data into a number of bins of a fixed width, and approximates the probabilities by the frequencies of occurrence. This simple approach has the drawback that the mutual information is systematically overestimated [30]. A more sophisticated option uses adaptive partitioning, where the bin size in the partition depends on the density of data points. This is the case of the classic algorithm by Fraser and Swinney [31], which manages to improve the estimations although at the cost of increasing the computation times considerably. A more efficient version of this method was presented in [32], together with a comparison of alternative numerical algorithms. Another computationally demanding option is to use kernel density estimation for estimating the probability density *p*(*x*), which can then be applied to estimation of mutual information [33]. Recently, Hausser and Strimmer [34] presented a procedure for the effective estimation of entropy and mutual information from small sample data, and demonstrated its application to the inference of high-dimensional gene association networks. More details about the influence of the choice of estimators of mutual information on the network inference problem can be found in [35,36], including numerical comparisons between several methods.

Another issue related to estimation of mutual information is the determination of a threshold to distinguish interaction from non-interaction. One solution is given by the minimum description length (MDL) principle [37], which states that, given a dataset and several candidate models, one should choose the model that provides the shortest encoding of the data. The MDL principle seeks to achieve a good trade-off between model complexity and accuracy of data fitting. It is similar to other criteria for model selection, such as the popular Akaike (AIC) and Bayesian information criterion (BIC). Like the BIC, the MDL takes into account the sample size, and minimizes both the model coding length and the data coding length.

We finish this section mentioning that a discussion of some issues concerning the definition of multivariate dependence has been presented in [38]. The aim of the analysis was to clarify the concept of dependence among different variables, in order to be able to distinguish between independent (additive) and cooperative (multiplicative) regulation.

### 2.3. Generalizations of Information Theory

In the 1960s Marko proposed a generalization of Shannon's information theory called bidirectional information theory [39,40]. Its aim was to distinguish the direction of information flow, which was considered necessary to describe generation and processing of information by living beings. The concept of Directed Transinformation (DTI) was introduced as extension of mutual information (which Shannon called transinformation). Let us consider two entities *M*_1_ and *M*_2_, with *X* being a symbol of *M*_1_ and *Y* of *M*_2_. Then the directed transinformation from *M*_1_ to *M*_2_ is

(13) $$T_{12}=lim_{n\to \infty }\;\left\{-\log\frac{p(X|X_{n})}{p(X|X_{n}Y_{n})}\right\}$$

where *p*(*X*∣*X_n_*) represents the conditional probability for the occurrence of *X* when *n* previous symbols *X_n_* of the own process are known, and *p*(*X*∣*X_n_Y_n_*) is the conditional probability for the occurrence of *X* when *n* previous symbols *X_n_* of the own process as well as of the other process *Y_n_* are known. The directed transinformation from *M*_2_ to *M*_1_ is defined in the same way, replacing *X* with *Y* and vice versa. The sum of both transinformations equals Shannon's transinformation or mutual information, that is:

(14) $$I=T_{12}+T_{21}$$

Marko's work was continued two decades later by Massey [41], who defined the directed information *I*(*X^N^* → *Y^N^*) from a sequence *X^N^* to a sequence *Y^N^* as a slight modification of the directed transinformation:

(15) $$I(X^{N}\to Y^{N})=\sum_{n=1}^{N}I(X^{n};Y_{n}|Y^{n-1})$$

If no feedback between *Y* and *X* is present, then the directed information and the traditional mutual information are equal, *I*(*X^N^* → *Y^N^*) = *I*(*X^N^*; *Y^N^*).

Another generalization of Shannon entropy is the concept of nonextensive entropy. Shannon entropy (also called Boltzmann–Gibbs entropy, which we denote here as *H_BG_*) agrees with standard statistical mechanics, a theory that applies to a large class of physical systems: those for which ergodicity is satisfied at the microscopic dynamical level. Standard statistical mechanics is extensive, that is, it assumes that, for a system *S* consisting of *N* independent subsystems *S*_1_, …, *S_N_*, it holds that $H_{BG}(S)=\sum_{i=1}^{N}H_{BG}(S_{i})$. This property is a result of the short-range nature of the interactions typically considered (think, for example, of the entropy of two subsets of an ideal gas). However, there are many systems where long-range interactions exist, and thus violate this hypothesis—a fact not always made explicit in the literature. To overcome this limitation, in 1988 Constantino Tsallis [42] proposed the following generalization of the Boltzmann–Gibbs entropy:

(16) $$H_{q}(X)=-k\frac{1-\sum_{i}^{\omega }p_{i}{\left(x\right)}^{q}}{1-q}$$

where *k* is a positive constant that sets the dimension and scale, *p_i_* are the probabilities associated with the *ω* distinct configurations of the system, and *q* ∈ ℜ is the so-called entropic parameter, which characterizes the generalization. The entropic parameter characterizes the degree of nonextensivity, which in the limit *q* → 1 recovers $H_{q=1}=-k\sum_{i}^{\omega }{\text{p}}_{i}\log p_{i}$, with *k* = *k_B_*, the Boltzmann constant. The generalized entropy *H_q_* is the basis of what has been called non-extensive statistical mechanics, as opposed to the standard statistical mechanics based on *H_BG_*. Indeed, *H_q_* is non-extensive for systems without correlations; however, for complex systems with long-range correlations the reverse is true: *H_BG_* is non-extensive and is not an appropriate entropy measure, while *H_q_* becomes extensive [43]. It has been suggested that the degree of nonextensivity can be used as a measure of complexity [44]. Scale-free networks [45,46] are an example of systems for which *H_q_* is extensive and *H_BG_* is not. Scale-free networks are characterized by the fact that their vertex connectivities follow a scale-free power-law distribution. It has been recognized that many complex systems from different areas—technological, social, and biological—are of this type. For these systems, it has been suggested that it is more meaningful to define the entropy in the form of Equation (16) instead of Equation (3). By defining the *q*-logarifhm function as $\ln_{q}\;(x)=\frac{x^{1-q}-1}{1-q}$, the nonextensive entropy can be expressed in a similar form as the Boltzmann–Gibbs entropy, Equation (3):

(17) $$H_{q}\;(X)=-\sum_{x}p(x)\;\ln_{q}\;p(x)=\sum_{x}\frac{p(x)-p{\left(x\right)}^{q}}{q-1}$$

and analogously one can define nonextensive versions of conditional entropy or mutual information.

## 3. Review of Network Inference Methods

### 3.1. Detecting Interactions: Correlations and Mutual Information

Early examples of techniques based on mutual information in a biological context can be found in [47], where it was used to determine eukaryotic protein coding regions, and [48], where it was applied for analyzing covariation of mutations in the V3 loop of the HIV-1 envelope protein. Since then many more examples have followed, with the first applications in network inference appearing in the second half of the 1990s. Specifically, in the 1998 Pacific Symposium on Biocomputing two methods for reverse engineering gene networks based on mutual information were presented. The REVEAL [49] algorithm used Boolean (on/off) models of gene networks and inferred interactions from mutual information. It was implemented in C and tested on synthetic data, with good results reported for a network of 50 elements and 3 inputs per element. In another contribution from the same symposium [50] mutual information was normalized as $I_{\text{Norm}}(X,Y)=\frac{I(X,Y)}{\max(H(X),H(Y))}$, and a distance matrix was then defined as *d^M^*(*X*,*Y*) = 1 − *I_N_m__*(*X*, *Y*). The distance matrix was used to find correlated patterns of gene expression from time series data. The normalization presents two advantages: the value of the distance *d* is between 0 and 1, and *d*(*X_i_*, *X_i_*) = 0.

Two years later, Butte *et al.* [51] proposed a technique for finding functional genomic clusters in RNA expression data, called mutual information relevance networks. Pair-wise mutual information between genes was calculated as in Equation (11), and it was hypothesized that associations with high mutual information were biologically related. Simultaneously, the same group published a related method [52] that used the correlation coefficient *r* Equation (1) instead of mutual information. The method, known as relevance networks (RN), was used to discover functional relationships between RNA expression and chemotherapeutic susceptibility. In this work the similarity of patterns of features was rated using pair-wise correlation coefficients defined as ${\hat{r}}^{2}=\frac{r}{\text{abs}(r)}r^{2}$. Butte *et al.* mentioned a number of advantages of their method over previous ones. First, relevance networks are able to display nodes with varying degrees of cross-connectivity, while phylogenetic-type trees such as the aforementioned [50] can only link each feature to one other feature, without additional links. Second, phylogenetic-type trees cannot easily cluster different types of biological data. For example, they can cluster genes and anticancer agents separately, but do not easily determine associations between genes and anticancer agents. Third, clustering methods such as [50] may ignore genes whose expression levels are highly negatively correlated across cell lines; in contrast, in RN negative and positive correlations are treated in the same way and are used in clustering.

Pearson's correlation coefficient was also used in [53] to assemble a gene coexpression network, with the ultimate goal of finding genetic modules that are conserved across evolution. DNA microarray data from humans, flies, worms, and yeast were used, and 22,163 coexpression relationships were found. The predictions implied by some of the discovered links were experimentally confirmed, and cell proliferation functions were identified for several genes.

In [54] transcriptional gene networks in human and mouse were reverse-engineered using a simple mutual information approach, where the expression values were discretized into three bins. The relevance of this study is due to the massive datasets used: 20,255 gene expression profiles from human samples, from which 4,817,629 connections were inferred. Furthermore, a subset of not previously described protein–protein interactions was experimentally validated. For a discussion on the use of information theory to detect protein–protein interactions, see Section 3.1 of [55].

The aforementioned methods were developed mostly for gene expression data. In contrast, the next two techniques, Correlation Metric Construction (CMC) and Entropy Metric Construction (EMC), aimed at reverse engineering chemical reaction mechanisms, and used time series data (typically metabolic) of the concentration of the species present in the mechanism. In CMC [56] the time-lagged correlations between two species are calculated as *S_ij_*(*τ*) =< (*x_i_*(*t*) − *x̄_i_*)(*x_j_*(*t* + *τ*) − *x̄_j_*) >, where <> denotes the time average over all measurements, and *x̄_i_* is the time average of the concentration of the time series of species *i*. From these functions a correlation matrix *R*(*τ*) is calculated; its elements are $r_{ij}\;(\tau )=S_{ij}\;(\tau )/\sqrt{S_{ii}\;(\tau )S_{jj}\;(\tau )}$. Then the elements $d_{ij}^{\text{CMC}}$ of the distance matrix are obtained as $d_{ij}^{\text{CMC}}={\left(c_{ii}-2c_{ij}+c_{jj}\right)}^{1/2}=\sqrt{2(1-c_{ij})}$, where *c_ij_* = *max* |*r_ij_*(*τ*)|*_τ_*. Finally, Multidimensional Scaling (MDS) is applied to the distance matrix, yielding a configuration of points representing each of the species, which are connected by lines that are estimates for the connectivities of the species in the reactions. Furthermore, the temporal ordering of the correlation maxima provides an indication of the causality of the reactions. CMC was first tested on a simulated chemical reaction mechanism [56], and was later successfully applied to the reconstruction of the glycolytic pathway from experimental data [57]. More recently, it has been integrated in a systematic model building pipeline [58], which includes not only inference of the chemical network, but also data preprocessing, automatic model family generation, model selection and statistical analysis.

The Entropy Metric Construction method, EMC [25,26], is a modification of CMC that replaces the correlation measures with entropy-based distances, $\frac{e^{H(X,Y)}}{e^{H(X)}e^{H(Y)}}=e^{H(X,Y)-H(X)-H(Y)}=e^{-I(X,Y)}$. The EMC correlation distance is the minimum regardless of $\tau :d_{ij}^{\text{EMC}}=\min_{\tau }e^{-I(X,Y)}$. If the correlation is Gaussian, usually $d_{ij}^{\text{CMC}}\approx d_{ij}^{\text{EMC}}$. Originally, EMC was applied to an artificial reaction mechanism with pseudo-experimental, for which it was reported to outperform CMC. Recently [59] it has been tested with the same glycolytic pathway reconstructed by CMC [57], with both methods yielding similar results.

Recently, CMC/EMC has inspired a method [60] that combines network inference by time-lagged correlation and estimation of kinetic parameters with a maximum likelihood approach. It was applied to a test case from pharmacokinetics: the deduction of the metabolic pathway of gemcitabine, using synthetic and experimental data.

The empirical distance correlation (DCOR) was presented in [61,62]. Given a random sample of *n* random vectors (*X*, *Y*), Euclidean distance matrices are calculated as *a_kl_* = |*X_k_* − *X_l_*|, *b_kl_* = |*Y_k_* − *Y_l_*|. Define ${\bar{a}}_{k•}=\frac{1}{n}\sum_{l=1}^{n}a_{kl}$, ${\bar{a}}_{•l}=\frac{1}{n}\sum_{k=1}^{n}a_{kl}$, ${\bar{a}}_{••}=\frac{1}{n^{2}}\sum_{k,l=1}^{n}a_{kl}$, *A_kl_* = *a_kl_* − *ā_k_*_•_ − *ā*_•_*_l_* + *ā*_••_, and similarly for *B_kl_*. Then the empirical distance covariance *ν_n_*(*X*, *Y*) is the nonnegative quantity defined by

(18) $$\nu_{n}^{2}(X,Y)=\frac{1}{n^{2}}\sum_{k,l=1}^{n}A_{kl}B_{kl}$$

Similarly, $\nu_{n}(X)=\nu_{n}(X,X)=\frac{1}{n^{2}}\sum_{k,l=1}^{n}A_{kl}^{2}$, and the distance correlation DCOR = *R_n_*(*X*, *Y*) is the square root of

(19) $$R_{n}^{2}(X,Y)=\{\begin{array}{ll} \frac{\nu_{n}^{2}(X,Y)}{\sqrt{\nu_{n}^{2}(X)\nu_{n}^{2}(Y)}}, & \nu_{n}^{2}(X)\nu_{n}^{2}(Y)>0\\ 0, & \nu_{n}^{2}(X)\nu_{n}^{2}(Y)=0 \end{array}$$

Unlike the classical definition of correlation, distance correlation is zero only if the random vectors are independent. Furthermore, it is defined for X and Y in arbitrary dimensions, rather than to univariate quantities. DCOR is a good example of a method that has gained recognition inside a research community (statistics) but whose merits have hardly become known to scientists working on other areas (such as the applied biological sciences). Some recent exceptions have recently appeared. In [63] it was used for the detection of long-range concerted motion in proteins. In a study concerning mortality [64], significant distance correlations were found between death ages, lifestyle factors, and family relationships. As for applications in network inference, [65] compared eight statistical measures, including distance covariance, evaluating their performance in gene association problems (the other measures being Spearman rank correlation, Weighted Rank Correlation, Kendall, Hoeffding's D measure, Theil–Sen, Rank Theil–Sen, and Pearson). Interestingly, the least efficient methods turned out to be Pearson and distance covariance.

The Maximal Information Coefficient (MIC) is another recently proposed measure of association between variables [66]. It was designed with the goal of assigning similar values to equally noisy relationships, independently of the type of association, a property termed “equitability”. The main idea behind MIC is that if two variables (*X*, *Y*) are related, their relationship can be encapsulated by a grid that partitions the data in the scatter plot. Thus, all possible grids are explored (up to a maximal resolution that depends on the sample size) and for each m-by-n grid the largest possible mutual information *I*(*X*,*Y*) is computed. Then the mutual information values are normalized between 0 and 1, ensuring a fair comparison between grids of different dimensions. The MIC measure is defined as the maximum of the normalized mutual information values [67]:

(20) $$\text{MIC}\;(X,Y)=\max_{|X||Y|<B}\frac{I\;(X,Y)}{\log\;(\min(|X|,|Y|))}$$

where |*X*| and |*Y*| are the number of bins for each variable and *B* the maximal resolution. This methodology has been applied to data sets in global health, gene expression, major-league baseball, and the human gut microbiota [66], demonstrating its ability for identifying known and novel relationships.

The claims about MIC's performance expressed in the original publication [66] have generated some criticism. In a comment posted on the publication web site, Simon and Tibshirani reminded that, since there is “no free lunch” in Statistics, tests designed to have high power against all alternatives have low power in many important situations. Hence, the fact that MIC has no preference for some alternatives over others (equitability) can be counterproductive in many cases. Simon and Tibshirani reported simulation results showing that MIC has lower power than DCOR for most relationships, and that in some cases MIC is less powerful than Pearson correlation as well. These deficiencies would indicate that MIC will produce many false positives in large scale problems, and that the use of the distance correlation measure is more advisable. In a similar comment, Gorfine *et al.* opposed the claim that non-equitable methods are less practical for data exploration, arguing that both DCOR and their own HHG method [68] are more powerful than the test based on MIC. At the moment of writing this article, the debate about the concept of equitability and its relation to mutual information and the MIC is very active at the arXiv website, with opposite views such as the ones expressed in [67,69].

Recently, the nonextensive entropy proposed by Tsallis has also been used in the context of reverse-engineering gene networks [70]. Given some temporal data, the method fixes a gene target *x_i_* and looks for the group of genes *g* that minimizes the nonextensive conditional entropy for a fixed *q*:

(21) $$H_{q}(x_{i}|g)=-\sum_{g=1}^{m}p(g)\frac{(1-\sum_{x_{i}}p{\left(x_{i}|g\right)}^{q})}{1-q}$$

The reported results show an improvement on the inference accuracy by adopting nonextensive entropies instead of traditional entropies. The best computational results in terms of reduction of the number of false positives were obtained with the range of values 2.5 < *q* < 3.5, which corresponds to subextensive entropy. This claim stresses the importance of the additional tuning parameter, *q*, allowed by the Tsallis entropy. The fact that *q* has to be fixed a priori is a drawback for its use in reverse engineering applications, since it is unclear how to choose its value.

Finally, we discuss some methods that use the minimum description length principle (MDL) described in Subsection 2.2. MDL was applied in [71] to infer gene regulatory networks from time series data, reporting good results with both synthetic datasets and experimental data from *Drosophila melanogaster.* While that method eliminated the need for a user-defined threshold value, it introduced the need for a user-defined tuning parameter to balance the contributions of model and data coding lengths. To overcome this drawback, in [72] it was proposed to use as the description length a theoretical measure derived from a maximum likelihood model. This alternative was reported to improve the accuracy of reconstructions of Boolean networks. The same goal was pursued in [73], where a network inference method that included a predictive MDL criterion was presented. This approach incorporated not only mutual information, but also conditional mutual information.

### 3.2. Distinguishing between Direct and Indirect Interactions

A number of methods have been proposed that use information theoretic considerations to distinguish between direct and indirect interactions. The underlying idea is to establish whether the variation in a variable can be explained by the variations in a subset of other variables in the system.

The Entropy Reduction Technique, ERT [25,26], is an extension of EMC that outputs the list of species X* with which a given species Y reacts, in order of the reaction strength. The mathematical formulation stems from the observation that, if a variable Y is completely independent of a set of variables **X**, then *H*(*Y*∣**X**) = *H*(*Y*); otherwise *H*(*Y*∣**X**) < *H*(*Y*). The ERT algorithm is defined as follows [25]:

1. Given a species Y, start with X* = ⊘
2. Find X* : *H*(*Y*∣X*, X*) = *min_X_H*(*Y*∣X*,X*)
3. Set X* = {X*, X*}
4. Stop if *H*(*Y*∣X*, X*) = *H*(*Y*∣X*), or when all species except Y are already in X*; otherwise go to step 2

Intuitively, the method determines whether the nonlinear variation in a variable Y, as given by its entropy, is explainable by the variations of a subset—possibly all—of the other variables in the system, X*. It is done by iterating through cycles of adding a variable X* to X* that minimizes *H*(*Y*∣X*) until further additions do not decrease the entropy. This technique leads to an ordered set of variables that control the variation in Y. A methodology called MIDER (Mutual Information Distance and Entropy Reduction), which combines and extends features of the ERT and EMC techniques, has been recently developed and a MATLAB implementation is available as a free software toolbox [59].

The ARACNE method [74–76] is an information-theoretic algorithm for identifying transcriptional interactions between gene products, using microarray expression profile data. It consists of two steps. In the first step, the mutual information between pairs of genes is calculated as in Equation (11), and pairs that have a mutual information greater than a threshold *I*_0_ are identified as candidate interactions. This part is similar to the method of mutual information relevance networks [51]. In the second step, the Data Processing Inequality (DPI) is applied to discard indirect interactions. The DPI is a well known property of mutual information [28] that simply states that, if *X* → *Y* → *Z* forms a Markov chain, then *I*(*X*, *Y*) ≥ *I*(*X*, *Z*). The ARACNE algorithm examines each gene triplet for which all three MIs are greater than *I*_0_ and removes the edge with the smallest value. In this way, ARACNE manages to reduce the number of false positives, which is a limitation of mutual information relevance networks. Indeed, when tested on synthetic data, ARACNE outperformed relevance networks and Bayesian networks. ARACNE has also been applied to experimental data, with the first application being reverse engineering of regulatory networks in human B cells [74]. If time-course data is available, a version of ARACNE that considers time delays [77] can be used.

The definition of Conditional Mutual Information (12) clearly suggests its application for distinguishing between direct and indirect applications. This is the idea underlying the method proposed in [78], which was tested on artificial and real (melanoma) datasets. The so-called direct connectivity metric (DCM) was introduced as a measure of the confidence in the prediction that two genes *X* and *Y* were connected. The DCM is defined as the following product:

(22) $$\text{DCM}=I(X,Y)\cdot \min_{Z\in V_{-XY}}I(X,Y|Z)$$

where *min_Z_*_∈_*_V_*_−_*_XY_I*(*X*, *Y*∣*Z*) is the least conditional mutual information given any other gene Z. This method was compared with ARACNE and mutual information relevance networks [51] and was reported to outperform them for certain datasets.

The Context Likelihood of Relatedness technique, CLR [79] adds a correction step to the calculation of mutual information, comparing the value of the mutual information between a transcription factor *X* and a gene *Y* with the background distribution of mutual information for all possible interactions involving *X* or *Y.* In this way the network context of the interactions is taken into account. The main idea behind CLR is that the most probable interactions are not necessarily those with the highest MI scores, but those whose scores are significantly above the background distribution; the additional correction step helps to remove false correlations. CLR was validated [79] using *E coli* data and known regulatory interactions from RegulonDB, and compared with other methods: relevance networks [52], ARACNe [74], and Bayesian networks [80]. It was reported [79] that CLR demonstrated a precision gain of 36% relative to the next best performing algorithm. In [81] CLR was compared with a module-based algorithm, LeMoNe (Learning Module Networks), using expression data and databases of known transcriptional regulatory interactions for *E coli* and *S. cerevisiae.* It was concluded that module-based and direct methods retrieve distinct parts of the networks.

The Minimum Redundancy Networks technique (MRNET [82]) was developed for inferring genetic networks from microarray data. It is based on a previous method for feature selection in supervised learning called maximum relevance/minimum redundancy (MRMR [83–85]). Given an output variable Y and a set of possible input variables X, MRMR ranks the inputs according to a score that is the difference between the mutual information with the output variable Y (maximum relevance) and the average mutual information with the previously ranked variables (minimum redundancy). By doing this MRMR is intended to select, from the least redundant variables, those that have the highest mutual information with the target. Thus, direct interactions should be better ranked than indirect interactions. The MRNET method uses the MRMR principle in the context of network inference. Comparisons with ARACNE, relevance networks, and CLR were carried out using synthetically generated data, showing that MRNET is competitive with these methods. The R/Bioconductor package *minet* [86] includes the four methods mentioned before, which can be used with four different entropy estimators and several validation tools. A known limitation of algorithms based on forward selection—such as MRNET—is that their results strongly depend on the first variable selected. To overcome this limitation, an enhanced version named MRNETB was presented in [87]; it improves the original method by using a backward selection strategy followed by a sequential replacement.

A statistical learning strategy called three-way mutual information (MI3) was presented in [88]. It was designed to infer transcriptional regulatory networks from high throughput gene expression data. The procedure is in principle sufficiently general to be applied to other reverse engineering problems. Consider three variables *R*_1_, *R*_2_, and *T*, where *R*_1_ and *R*_2_ are possible “regulators” of the target variable, *T*. Then the MB metric is defined as

(23) $$\begin{array}{ll} MI3(T;R_{1},R_{2}) & =2I(T,(R_{1},R_{2}))-I(T,R_{1})-I(T,R_{2})\\ & =2H(R_{1},R_{2})+H(R_{1},T)+H(R_{2},T)-H(R_{1})-H(R_{2})-2H(R_{1},R_{2},T) \end{array}$$

Both MI3 and ERT try to detect higher order interactions and, for this purpose, they use scores calculated from entropies H(*), and 2- and 3- variable joint entropies, H(*,*) and H(*,*,*). MI3 was specifically designed to detect cooperative activity between two regulators in transcriptional regulatory networks, and it was reported to outperform other methods such as Bayesian networks, two-way mutual information and a discrete version of MI3. A method [89] exploiting three-way mutual information and CLR was the best scorer in the 2nd conference on Dialogue for Reverse Engineering Assessments and Methods (DREAM2) Challenge 5 (unsigned genome-scale network prediction from blinded microarray data) [90].

A similar measure, averaged three-way mutual information (AMD), was defined in [91] as

(24) $$I_{\text{ijk}}=I(Y_{j};X_{i})+I(Y_{k};X_{i})+I(Y_{j};Y_{k}|X_{i})-I(Y_{j};Y_{k})$$

where *X_i_* represents the target gene, and *Y_j_*, *Y_k_* are two regulators that may regulate *X_i_* cooperatively. The first two terms are the traditional mutual information. The third term represents the cooperative activity between *Y_j_* and *Y_k_*, and the fourth term ensures that *Y_j_* and *Y_k_* regulate *X_i_* directly (without regulation between *Y_j_* and *Y_k_*): if *Y_j_* regulates *X_i_* indirectly through *Y_k_*, both the third and fourth terms will increase, cancelling each other and not leading to an increase in *I_ijk_*. In [91] this score was combined with non-linear ordinary differential equation (ODE) modeling for inferring transcriptional networks from gene expression, using network-assisted regression. The resulting method was tested with synthetic data, reporting better performance than other algorithms (ARACNE, CLR, MRNET and SA-CLR). It was also applied to experimental data from *E. coli* and yeast, allowing to make new predictions.

The Inferelator [92] is another freely available method for network inference. It was designed for inferring genome-wide transcriptional regulatory interactions, using standard regression and model shrinkage techniques to model the expression of a gene or cluster of genes as a function of the levels of transcription factors and other influences. Its performance was demonstrated in [93], where the transcriptional regulatory network of *Halobacterium salinarum NRC-1* was reverse-engineered and its responses in 147 experiments were successfully predicted. Although the Inferelator is not itself based on mutual information, it has performed best when combined with MI methods [94]. Specifically, it has been used jointly with CLR.

Other methods have relied on correlation measures instead of mutual information for detecting indirect interactions. A method to construct approximate undirected dependency graphs from large-scale biochemical data using partial correlation coefficients was proposed in [95]. In a first step networks are built based on correlations between chemical species. The Pearson correlation coefficient (1) or the Spearman correlation coefficient may be chosen. The Spearman correlation coefficient is simply the Pearson correlation coefficient between the ranked variables, and measures how well the relationship between two variables can be described using a monotonic function. In a second step edges for which the partial correlation coefficient (2) falls below a certain threshold are eliminated. This procedure was tested both on artificial and on experimental data, and a software implementation was made available at the website.

In [96] partial correlation was used to reduce the number of candidate genes. In the partial correlation Equation (2) the Pearson correlation coefficient *r* was replaced by the Spearman correlation coefficient, since the latter was found to be more robust for detecting nonlinear relationships between genes. However, the authors acknowledged that the issue deserved further investigation.

In [97] both Pearson and Spearman correlation coefficients were tested; no practical differences were found between both measures. Furthermore, no clear differences were detected between linear (correlation) and nonlinear (mutual information) scores. For detecting indirect interactions, three different tools were used: partial correlation, conditional mutual information, and the data processing inequality, which were found to improve noticeably the performance of their non-conditioned counterparts. These results were obtained from artificially generated metabolic data.

### 3.3. Detecting Causality

Inferring the causality of an interaction is a complicated task, with deep theoretical implications. This topic has been extensively investigated by Pearl [98]. Philosophical considerations aside, from a practical view point we can intuitively assign a causal relation from A to B if A and B are correlated and A precedes B. Thus, causal interactions can be inferred if time series data is available.

It was already mentioned that CMC can determine directionality because it takes time series information into account, as shown in [57] for a glycolytic path. Another network reconstruction algorithm based on correlations was proposed in [99] to deduce directional connections based on gene expression measurements. Here the directionality came from the asymmetry of the conditional correlation matrix, which expressed the correlation between two genes given that one of them was perturbed. Another approach for causal correlations was presented in Opgen-Rhein and Strimmer [100]. Once the correlation network is obtained, a partial ordering of the nodes is established by multiple testing of the log-ratio of standardized partial variances. In this way a directed acyclic causal network is obtained as a subgraph of the original network. This method was validated using gene expression data of *Arabidopsis thaliana*.

Some methods based on mutual information have taken causality into account. One of them is EMC [26], which is essentially the same method as CMC with a different definition of distance. Another one is the already mentioned TimeDelay-ARACNE method [77]. The concept of directed information described in Section 2 has also been applied to the reconstruction of biological networks. In [101] it was used for reconstructing gene networks; the method was validated using small random networks and simulated data from the *E.Coli* network for flagella biosynthesis. It was reported that, for acyclic graphs with 7 or fewer genes with summation operations only, the method was able to infer all edges. In [102] directed information was used for finding interactions between transcription factor modules and target co-regulated genes. The validity of the approach was demonstrated using publicly available embryonic kidney and T-cell microarray datasets. DTInfer, an R-package for the inference of gene-regulatory networks from microarrays using directed information, was presented in [103]. It was tested on *E. coli* data, predicting five novel TF-target gene interactions; one of them was validated experimentally. Finally, directed information has also been used in a neuroscience context [104], for inferring causal relationships in ensemble neural spike train recordings.

### 3.4. Previous Comparisons

As mentioned in the Introduction, there are some publications where detailed analyses and comparisons of some of the methods reviewed here have been carried out. For example, in [17] the performance of some popular algorithms was tested under different conditions and on both synthetic and real data. Comparisons were twofold: on the one hand, conditional similarity measures like partial Pearson correlation (PPC), graphical Gaussian models (GGM), and conditional mutual information (CMI) were compared with Pearson correlation (PC) and mutual information (MI); on the other hand, linear measures (PC and PPC) were compared with non-linear ones (MI, CMI, and the Data Processing Inequality, DPI).

The differences and similarities of three other network inference algorithms—ARACNE, Context Likelihood of Relatedness (CLR), and MRNET—were studied in [35], where the influence of the entropy estimator was also taken into account. The performance of the methods was found to be dependent on the quality of the data: when complete and accurate measurements were available, the MRNET method combined with the Spearman correlation appeared to be the most effective. However, in the case of noisy and incomplete data, the best performer was CLR combined with Pearson correlation.

The same three inference algorithms, together with the Relevance Networks method (RN), were compared in [18], using network-based measures in combination with ensemble simulations. In [105] Emmert-Streib studied the influence of environmental conditions on the performance of five network inference methods, ARACNE, BC3NET [106], CLR, C3NET [107], and MRNET. Comparison of their results for three different conditions concluded that different statistical methods lead to comparable but condition-specific results. The tutorial [19] evaluated the performance of ARACNE, BANJO, NIR/MNI, and hierarchical clustering, using synthetic data. More recently, [20] compared four tools for inferring regulatory networks (ARACNE, BANJO, MIKANA, and SiGN-BN), applying them to new microarray datasets generated in human endothelial cells.

## 4. Conclusions, Successes and Challenges

A number of methods for inferring the connectivity of cellular networks has been reviewed in this article. Most of these methods, which have been published during the last two decades, adopt some sort of information theoretic approach for evaluating the probability of the interactions between network components. We have tried to review as many techniques as possible, surveying the literature from areas such as systems and computational biology, bioinformatics, molecular biology, microbiology, biophysics, physical and computational chemistry, physics, systems and process control, computer science, or statistics. Some methods were designed for specific purposes (e.g., reverse engineering gene regulatory networks), while others aim at a wider range of applications. We have attempted to give a unified treatment to methods from different backgrounds, clarifying their differences and similarities. When available, comparisons of their performances have been reported.

It has been shown that information theory provides a solid foundation for developing reverse engineering methodologies, as well as a framework to analyze and compare them. Concepts such as entropy or mutual information are of general applicability and make no assumptions about the underlying systems; for example, they do not require linearity or absence of noise. Furthermore, most information theoretic methods are scalable and can be applied to large-scale networks with hundreds or thousands of components. This gives them in some cases an advantage over other techniques that have higher computational cost, such as Bayesian approaches.

A conclusion of this review is that no single method outperforms the rest for all problems. There is “no free lunch”: methods that are carefully tailored to a particular application or dataset may yield better results than others when applied to that particular problem, but frequently perform worse when applied to different systems. Therefore, when facing a new problem it may be useful to try several methods. Interestingly, the results of the DREAM challenges show that community predictions are more reliable than individual predictions [108–110]; that is, the best option is to take into account the reconstructions provided by all the methods, as opposed to trusting only the best performing ones.

In the last fifteen years different information theoretic methods have been successfully applied to the reverse engineering of genetic networks. The resulting predictions about existing interactions have enabled the design of new experiments and the generation of hypotheses that were later confirmed experimentally, demonstrating the ability of computational modeling to provide biological insight. Another indication of the success of the information theoretic approach is that in recent years methods that combine mutual information with other techniques have been among the top performers in the DREAM reverse engineering challenges [94]. Success stories have appeared also regarding their application to reconstruction of chemical reaction mechanisms. One of the earliest was the validation of the CMC method, which was able to infer a significant part of the glycolytic path from experimental data.

Despite all the advances made in the last decades, the problem faced by these methods (inferring large-scale networks with nonlinear interactions from incomplete and noisy data) remains challenging. To progress towards that goal, several breakthroughs need to be achieved. A systematic way of determining causality that is valid for large-scale systems is still lacking. Computational and experimental procedures for identifying feedback loops and other complex structures are also needed. For these and other obstacles to be overcome, the future developments should be aware of the existing methodologies and build on their capabilities. We hope that this review will help researchers in that task.

## References

[1] Kitano H. (2001). Foundations of Systems Biology.

[2] Arkin A., Schaffer D. (2011). Network news: Innovations in 21st century systems biology. Cell.

[3] Gray R. (2009). Entropy and Information Theory.

[4] Shannon C. (1948). A mathematical theory of communication. Bell Syst. Tech. J..

[5] Quastler H. (1953). Information Theory in Biology.

[6] Bekey G., Beneken J. (1978). Identification of biological systems: A survey. Automatica.

[7] D'haeseleer P., Liang S., Somogyi R. (2000). Genetic network inference: From co-expression clustering to reverse engineering. Bioinformatics.

[8] Crampin E., Schnell S., McSharry P. (2004). Mathematical and computational techniques to deduce complex biochemical reaction mechanisms. Prog. Biophys. Mol. Biol..

[9] Ross J. (2008). Determination of complex reaction mechanisms. Analysis of chemical, biological and genetic networks. J. Phys. Chem. A.

[10] De Jong H. (2002). Modeling and simulation of genetic regulatory systems: A literature review. J. Comput. Biol..

[11] Cho K., Choo S., Jung S., Kim J., Choi H., Kim J. (2007). Reverse engineering of gene regulatory networks. IET Syst. Biol..

[12] Markowetz F., Spang R. (2007). Inferring cellular networks–A review. BMC Bioinform..

[13] Hecker M., Lambeck S., Toepfer S., van Someren E., Guthke R. (2009). Gene regulatory network inference: Data integration in dynamic models–A review. Biosystems.

[14] López-Kleine L., Leal L., López C. (2013). Biostatistical approaches for the reconstruction of gene co-expression networks based on transcriptomic data. Brief. Funct. Genomics.

[15] Koyutürk M. (2009). Algorithmic and analytical methods in network biology. WIREs Syst. Biol. Med..

[16] De Smet R., Marchal K. (2010). Advantages and limitations of current network inference methods. Nat. Rev. Microbiol..

[17] Soranzo N., Bianconi G., Altafini C. (2007). Comparing association network algorithms for reverse engineering of large-scale gene regulatory networks: Synthetic versus real data. Bioinformatics.

[18] Altay G., Emmert-Streib F. (2010). Revealing differences in gene network inference algorithms on the network level by ensemble methods. Bioinformatics.

[19] Bansal M., Belcastro V., Ambesi-Impiombato A., di Bernardo D. (2007). How to infer gene networks from expression profiles. Mol. Syst. Biol..

[20] Hurley D., Araki H., Tamada Y., Dunmore B., Sanders D., Humphreys S., Affara M., Imoto S., Yasuda K., Tomiyasu Y. (2012). Gene network inference and visualization tools for biologists: Application to new human transcriptome datasets. Nucleic Acids Res..

[21] Walter E., Pronzato L. (1997). Identification of parametric models from experimental data. Communications and Control Engineering Series.

[22] Ljung L. (1999). System Identification: Theory for the User.

[23] Galton F. (1886). Regression towards mediocrity in hereditary stature. J. Anthropol. Inst. Great Brit. Ire..

[24] Stigler S. (1989). Francis Galton's account of the invention of correlation. Stat. Sci..

[25] Samoilov M. (1997). Reconstruction and functional analysis of general chemical reactions and reaction networks. PhD thesis.

[26] Samoilov M., Arkin A., Ross J. (2001). On the deduction of chemical reaction pathways from measurements of time series of concentrations. Chaos.

[27] Linfoot E. (1957). An informational measure of correlation. Inf. Control.

[28] Cover T., Thomas J. (1991). Elements of Information Theory.

[29] Numata J., Ebenhöh O., Knapp E. (2008). Measuring correlations in metabolomic networks with mutual information. Genome Inform..

[30] Steuer R., Kurths J., Daub C., Weise J., Selbig J. (2002). The mutual information: Detecting and evaluating dependencies between variables. Bioinformatics.

[31] Fraser A., Swinney H. (1986). Independent coordinates for strange attractors from mutual information. Phys. Rev. A.

[32] Cellucci C., Albano A., Rapp P. (2005). Statistical validation of mutual information calculations: Comparison of alternative numerical algorithms. Phys. Rev. E.

[33] Moon Y., Rajagopalan B., Lall U. (1995). Estimation of mutual information using kernel density estimators. Phys. Rev. E.

[34] Hausser J., Strimmer K. (2009). Entropy inference and the James-Stein estimator, with application to nonlinear gene association networks. J. Mach. Learn. Res..

[35] Olsen C., Meyer P., Bontempi G. (2009). On the impact of entropy estimation on transcriptional regulatory network inference based on mutual information. EURASIP J. Bioinform. Syst. Biol..

[36] De Matos Simoes R., Emmert-Streib F. (2011). Influence of statistical estimators of mutual information and data heterogeneity on the inference of gene regulatory networks. PLoS One.

[37] Rissanen J. (1978). Modeling by shortest data description. Automatica.

[38] Margolin A., Wang K., Califano A., Nemenman I. (2010). Multivariate dependence and genetic networks inference. IET Syst. Biol..

[39] Marko H. (1967). Information theory and cybernetics. IEEE Spectrum.

[40] Marko H. (1973). The bidirectional communication theory–a generalization of information theory. IEEE Trans. Commun..

[41] Massey J. Causality, feedback and directed information.

[42] Tsallis C. (1988). Possible generalization of Boltzmann-Gibbs statistics. J. Stat. Phys..

[43] Tsallis C., Gell-Mann M., Sato Y. (2005). Asymptotically scale-invariant occupancy of phase space makes the entropy Sq extensive. Proc. Natl. Acad. Sci. USA.

[44] Tsallis C. (2002). Entropic nonextensivity: A possible measure of complexity. Chaos Soliton Fract..

[45] Barabási A., Albert R. (1999). Emergence of scaling in random networks. Science.

[46] Barabási A. (2009). Scale-free networks: A decade and beyond. Science.

[47] Farber R., Lapedes A., Sirotkin K. (1992). Determination of eukaryotic protein coding regions using neural networks and information theory. J. Mol. Biol..

[48] Korber B., Farber R., Wolpert D., Lapedes A. (1993). Covariation of mutations in the V3 loop of human immunodeficiency virus type 1 envelope protein: an information theoretic analysis. Proc. Natl. Acad. Sci. USA.

[49] Liang S., Fuhrman S., Somogyi R. REVEAL, a general reverse engineering algorithm for inference of genetic network architectures.

[50] Michaels G., Carr D., Askenazi M., Fuhrman S., Wen X., Somogyi R. Cluster analysis and data visualization of large scale gene expression data.

[51] Butte A., Kohane I. Mutual information relevance networks: Functional genomic clustering using pairwise entropy measurements.

[52] Butte A., Tamayo P., Slonim D., Golub T., Kohane I. (2000). Discovering functional relationships between RNA expression and chemotherapeutic susceptibility using relevance networks. Proc. Natl. Acad. Sci. USA.

[53] Stuart J., Segal E., Koller D., Kim S. (2003). A gene-coexpression network for global discovery of conserved genetic modules. Science.

[54] Belcastro V., Siciliano V., Gregoretti F., Mithbaokar P., Dharmalingam G., Berlingieri S., Iorio F., Oliva G., Polishchuck R., Brunetti-Pierri N. (2011). Transcriptional gene network inference from a massive dataset elucidates transcriptome organization and gene function. Nucleic Acids Res..

[55] Adami C. (2004). Information theory in molecular biology. Phys. Life Rev..

[56] Arkin A., Ross J. (1995). Statistical Construction of chemical reaction mechanisms from measured time-series. J. Phys. Chem..

[57] Arkin A., Shen P., Ross J. (1997). A test case of correlation metric construction of a reaction pathway from measurements. Science.

[58] Wahl S., Haunschild M., Oldiges M., Wiechert W. (2006). Unravelling the regulatory structure of biochemical networks using stimulus response experiments and large-scale model selection. Syst. Biol..

[59] Villaverde A.F., Ross J., Morán F., Banga J.R. (2013). MIDER: Network inference with Mutual Information Distance and Entropy Reduction. http://www.iim.csic.es/gingproc/mider.html/.

[60] Lecca P., Morpurgo D., Fantaccini G., Casagrande A., Priami C. (2012). Inferring biochemical reaction pathways: The case of the gemcitabine pharmacokinetics. BMC Syst. Biol..

[61] Székely G., Rizzo M., Bakirov N. (2007). Measuring and testing dependence by correlation of distances. Ann. Stat..

[62] Szekely G., Rizzo M. (2009). Brownian distance correlation. Ann. Appl. Stat..

[63] Roy A., Post C. (2012). Detection of long-range concerted motions in protein by a distance covariance. J. Chem. Theory Comput..

[64] Kong J., Klein B., Klein R., Lee K., Wahba G. (2012). Using distance correlation and SS-ANOVA to assess associations of familial relationships, lifestyle factors, diseases, and mortality. Proc. Natl. Acad. Sci. USA.

[65] Kumari S., Nie J., Chen H., Ma H., Stewart R., Li X., Lu M., Taylor W., Wei H. (2012). Evaluation of gene association methods for coexpression network construction and biological knowledge discovery. PLoS One.

[66] Reshef D., Reshef Y., Finucane H., Grossman S., McVean G., Turnbaugh P., Lander E., Mitzenmacher M., Sabeti P. (2011). Detecting novel associations in large data sets. Science.

[67] Kinney J., Atwal G. (2013). Equitability, mutual information, and the maximal information coefficient. arXiv.

[68] Heller R., Heller Y., Gorfine M. (2012). A consistent multivariate test of association based on ranks of distances. arXiv.

[69] Reshef D., Reshef Y., Mitzenmacher M., Sabeti P. (2013). Equitability analysis of the maximal information coefficient, with comparisons. arXiv.

[70] Lopes F., de Oliveira E., Cesar R. (2011). Inference of gene regulatory networks from time series by Tsallis entropy. BMC Syst. Biol..

[71] Zhao W., Serpedin E., Dougherty E.R. (2006). Inferring gene regulatory networks from time series data using the minimum description length principle. Bioinformatics.

[72] Dougherty J., Tabus I., Astola J. (2008). Inference of gene regulatory networks based on a universal minimum description length. EURASIP J. Bioinform. Syst. Biol..

[73] Chaitankar V., Ghosh P., Perkins E., Gong P., Deng Y., Zhang C. (2010). A novel gene network inference algorithm using predictive minimum description length approach. BMC Syst. Biol..

[74] Basso K., Margolin A., Stolovitzky G., Klein U., Dalla-Favera R., Califano A. (2005). Reverse engineering of regulatory networks in human B cells. Nat. Genet..

[75] Margolin A., Wang K., Lim W., Kustagi M., Nemenman I., Califano A. (2006). Reverse engineering cellular networks. Nat. Protoc..

[76] Margolin A., Nemenman I., Basso K., Wiggins C., Stolovitzky G., Favera R., Califano A. (2006). ARACNE: An algorithm for the reconstruction of gene regulatory networks in a mammalian cellular context. BMC Bioinform..

[77] Zoppoli P., Morganella S., Ceccarelli M. (2010). TimeDelay-ARACNE: Reverse engineering of gene networks from time-course data by an information theoretic approach. BMC Bioinform..

[78] Zhao W., Serpedin E., Dougherty E. (2008). Inferring connectivity of genetic regulatory networks using information-theoretic criteria. IEEE ACM Trans. Comput. Biol. Bioinformatics.

[79] Faith J., Hayete B., Thaden J., Mogno I., Wierzbowski J., Cottarel G., Kasif S., Collins J., Gardner T. (2007). Large-scale mapping and validation of Escherichia coli transcriptional regulation from a compendium of expression profiles. PLoS Biol..

[80] Friedman N., Linial M., Nachman I., Pe'er D. (2000). Using Bayesian networks to analyze expression data. J. Comput. Biol..

[81] Michoel T., de Smet R., Joshi A., van de Peer Y., Marchal K. (2009). Comparative analysis of module-based versus direct methods for reverse-engineering transcriptional regulatory networks. BMC Syst. Biol..

[82] Meyer P., Kontos K., Lafitte F., Bontempi G. (2007). Information-theoretic inference of large transcriptional regulatory networks. EURASIP J. Bioinform. Syst. Biol..

[83] Tourassi G., Frederick E., Markey M., Floyd J. (2001). Application of the mutual information criterion for feature selection in computer-aided diagnosis. Med. Phys..

[84] Peng H., Long F., Ding C. (2005). Feature selection based on mutual information: Criteria of max-dependency, max-relevance, and min-redundancy. IEEE Trans. Pattern Anal. Mach. Intell..

[85] Ding C., Peng H. (2005). Minimum redundancy feature selection from microarray gene expression data. J. Bioinform. Comput. Biol..

[86] Meyer P., Lafitte F., Bontempi G. (2008). Minet: A R/Bioconductor package for inferring large transcriptional networks using mutual information. BMC Bioinform..

[87] Meyer P., Marbach D., Roy S., Kellis M. Information-theoretic inference of gene networks using backward elimination.

[88] Luo W., Hankenson K., Woolf P. (2008). Learning transcriptional regulatory networks from high throughput gene expression data using continuous three-way mutual information. BMC Bioinform..

[89] Watkinson J., Liang K., Wang X., Zheng T., Anastassiou D. (2009). Inference of regulatory gene interactions from expression data using three-way mutual information. Ann. N.Y. Acad. Sci..

[90] Stolovitzky G., Prill R., Califano A. (2009). Lessons from the DREAM2 Challenges. Ann. N.Y. Acad. Sci..

[91] Wang X., Qi Y., Jiang Z. (2011). Reconstruction of transcriptional network from microarray data using combined mutual information and network-assisted regression. IET Syst. Biol..

[92] Bonneau R., Reiss D., Shannon P., Facciotti M., Hood L., Baliga N., Thorsson V. (2006). The Inferelator: An algorithm for learning parsimonious regulatory networks from systems-biology data sets. de novo. Genome Biol..

[93] Bonneau R., Facciotti M., Reiss D., Schmid A., Pan M., Kaur A., Thorsson V., Shannon P., Johnson M., Bare J. (2007). A predictive model for transcriptional control of physiology in a free living cell. Cell.

[94] Greenfield A., Madar A., Ostrer H., Bonneau R. (2010). DREAM4: Combining genetic and dynamic information to identify biological networks and dynamical models. PLoS One.

[95] De La Fuente A., Bing N., Hoeschele I., Mendes P. (2004). Discovery of meaningful associations in genomic data using partial correlation coefficients. Bioinformatics.

[96] Bing N., Hoeschele I. (2005). Genetical genomics analysis of a yeast segregant population for transcription network inference. Genetics.

[97] Çakır T., Hendriks M., Westerhuis J., Smilde A. (2009). Metabolic network discovery through reverse engineering of metabolome data. Metabolomics.

[98] Pearl J. (2010). An introduction to causal inference. Int. J. Biostat..

[99] Rice J., Tu Y., Stolovitzky G. (2005). Reconstructing biological networks using conditional correlation analysis. Bioinformatics.

[100] Opgen-Rhein G., Strimmer K. (2007). From correlation to causation networks: A simple approximate learning algorithm and its application to high-dimensional plant gene expression data. BMC Syst. Biol..

[101] Mathai P., Martins N., Shapiro B. On the detection of gene network interconnections using directed mutual information.

[102] Rao A., Hero A., States D., Engel J. (2008). Using directed information to build biologically relevant influence networks. J. Bioinform. Comput. Biol..

[103] Kaleta C., Göhler A., Schuster S., Jahreis K., Guthke R., Nikolajewa S. (2010). Integrative inference of gene-regulatory networks in Escherichia coli using information theoretic concepts and sequence analysis. BMC Syst. Biol..

[104] Quinn C., Coleman T., Kiyavash N., Hatsopoulos N. (2011). Estimating the directed information to infer causal relationships in ensemble neural spike train recordings. J. Comput. Neurosci..

[105] Emmert-Streib F. (2013). Influence of the experimental design of gene expression studies on the inference of gene regulatory networks: Environmental factors. PeerJ.

[106] De Matos Simoes R., Emmert-Streib F. (2012). Bagging statistical network inference from large-scale gene expression data. PLoS One.

[107] Altay G., Emmert-Streib F. (2010). Inferring the conservative causal core of gene regulatory networks. BMC Syst. Biol..

[108] Marbach D., Prill R., Schaffter T., Mattiussi C., Floreano D., Stolovitzky G. (2010). Revealing strengths and weaknesses of methods for gene network inference. Proc. Natl. Acad. Sci. USA.

[109] Prill R., Saez-Rodriguez J., Alexopoulos L., Sorger P., Stolovitzky G. (2011). Crowdsourcing network inference: The DREAM predictive signaling network challenge. Sci. Signal..

[110] Marbach D., Costello J., Küffner R., Vega N., Prill R., Camacho D., Allison K., Kellis M., Collins J., Stolovitzky G. (2012). Wisdom of crowds for robust gene network inference. Nat. Methods.

